# Supplementary material for: The inflammatory microenvironment of the lung at the time of infection governs innate control of SARS-CoV-2 replication
Source: bioRxiv. 2024 Mar 27:2024.03.27.586885. Preprint. [Version 1] doi: 10.1101/2024.03.27.586885 (PMC10996686; doi:10.1101/2024.03.27.586885)

bioRxiv preprint doi: https://doi.org/10.1101/2024.03.27.586885; this version posted March 27, 2024. The copyright holder for this preprint (which was not certified by peer review) is the author/funder. This article is a US Government work. It is not subject to copyright under 17 USC 105 and is also made available for use under a CC0 license.

Baker et al. Figure S1

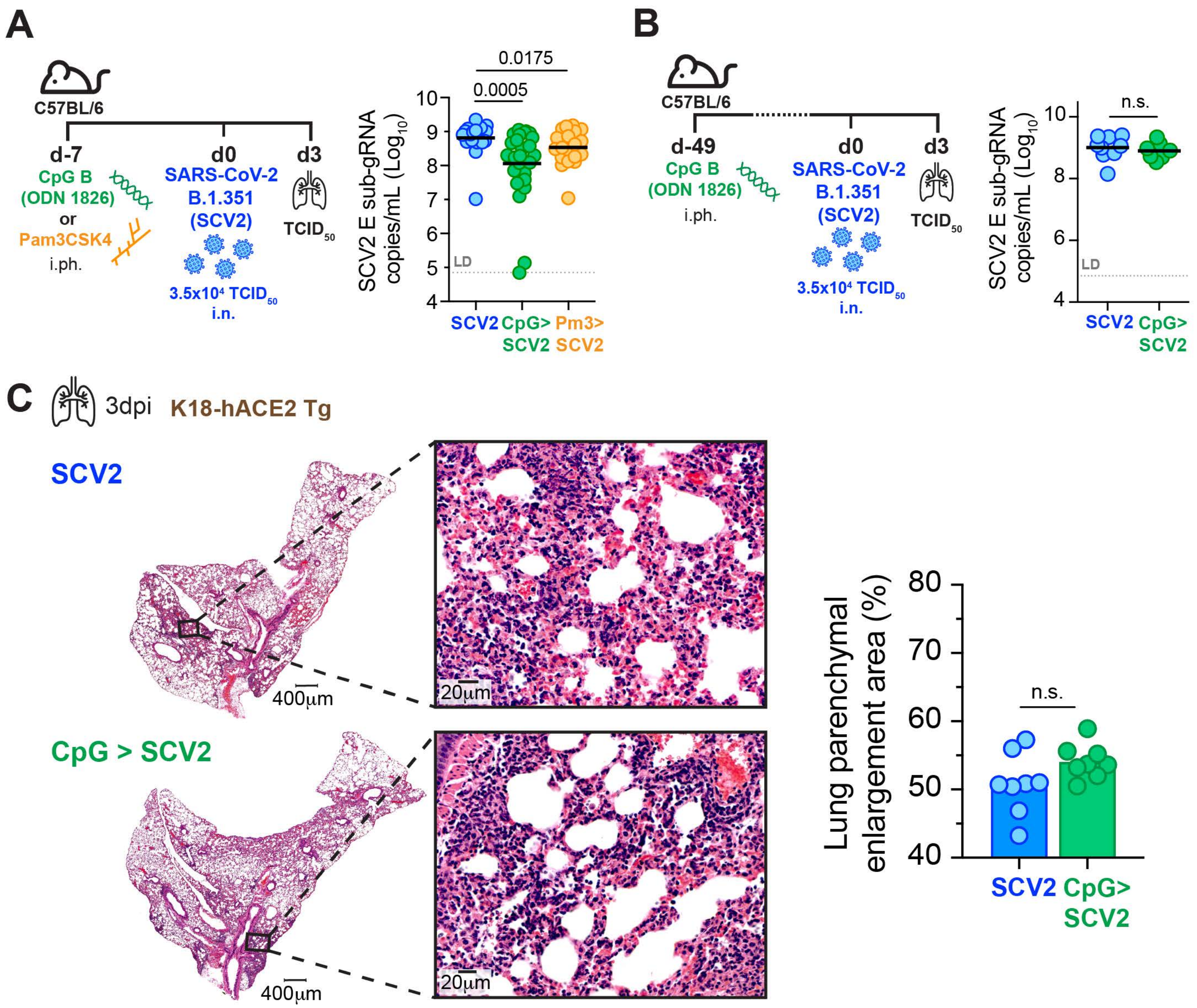

bioRxiv preprint doi: https://doi.org/10.1101/2024.03.27.586885; this version posted March 27, 2024. The copyright holder for this preprint (which was not certified by peer review) is the author/funder. This article is a US Government work. It is not subject to copyright under 17 USC 105 and is also made available for use under a CC0 license.

Baker et al. Figure S2

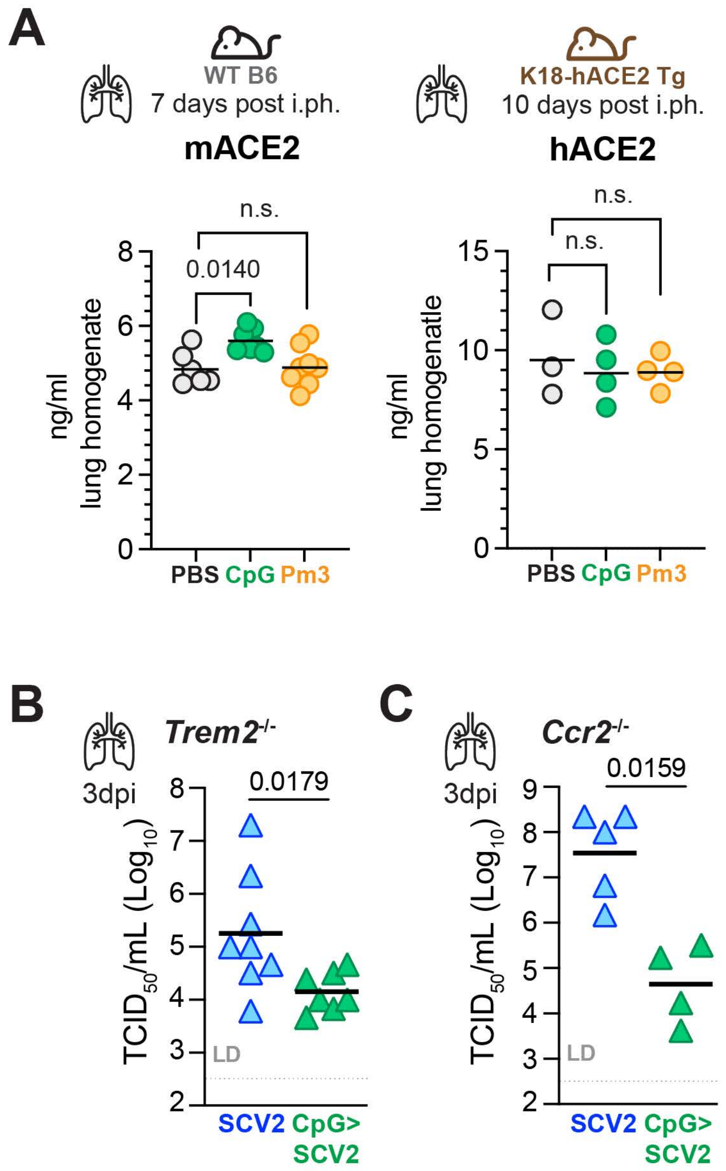

bioRxiv preprint doi: https://doi.org/10.1101/2024.03.27.586885; this version posted March 27, 2024. The copyright holder for this preprint (which was not certified by peer review) is the author/funder. This article is a US Government work. It is not subject to copyright under 17 USC 105 and is also made available for use under a CC0 license.

Baker et al. Figure S3

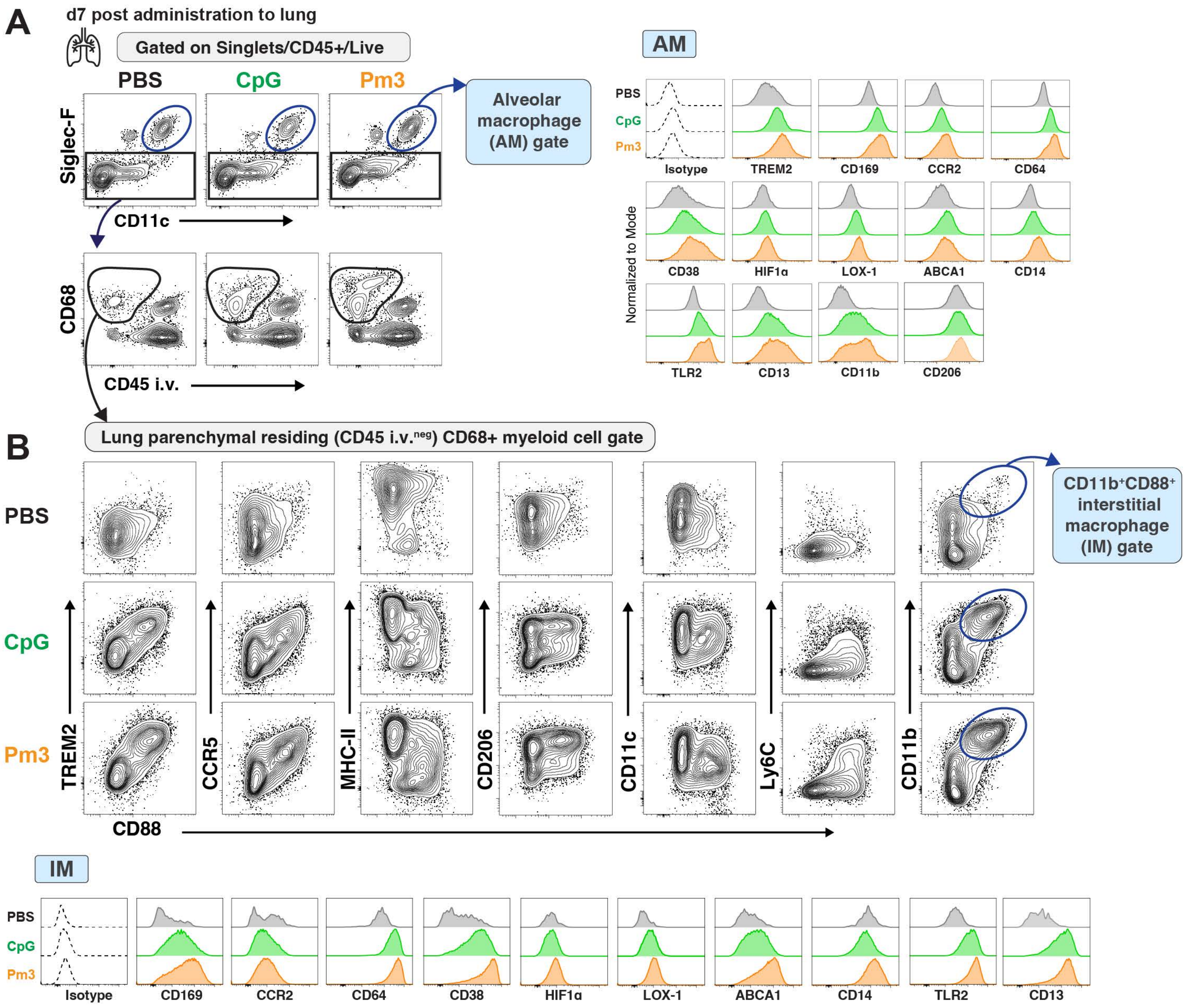

bioRxiv preprint doi: https://doi.org/10.1101/2024.03.27.586885; this version posted March 27, 2024. The copyright holder for this preprint (which was not certified by peer review) is the author/funder. This article is a US Government work. It is not subject to copyright under 17 USC 105 and is also made available for use under a CC0 license.

Baker et al. Figure S4

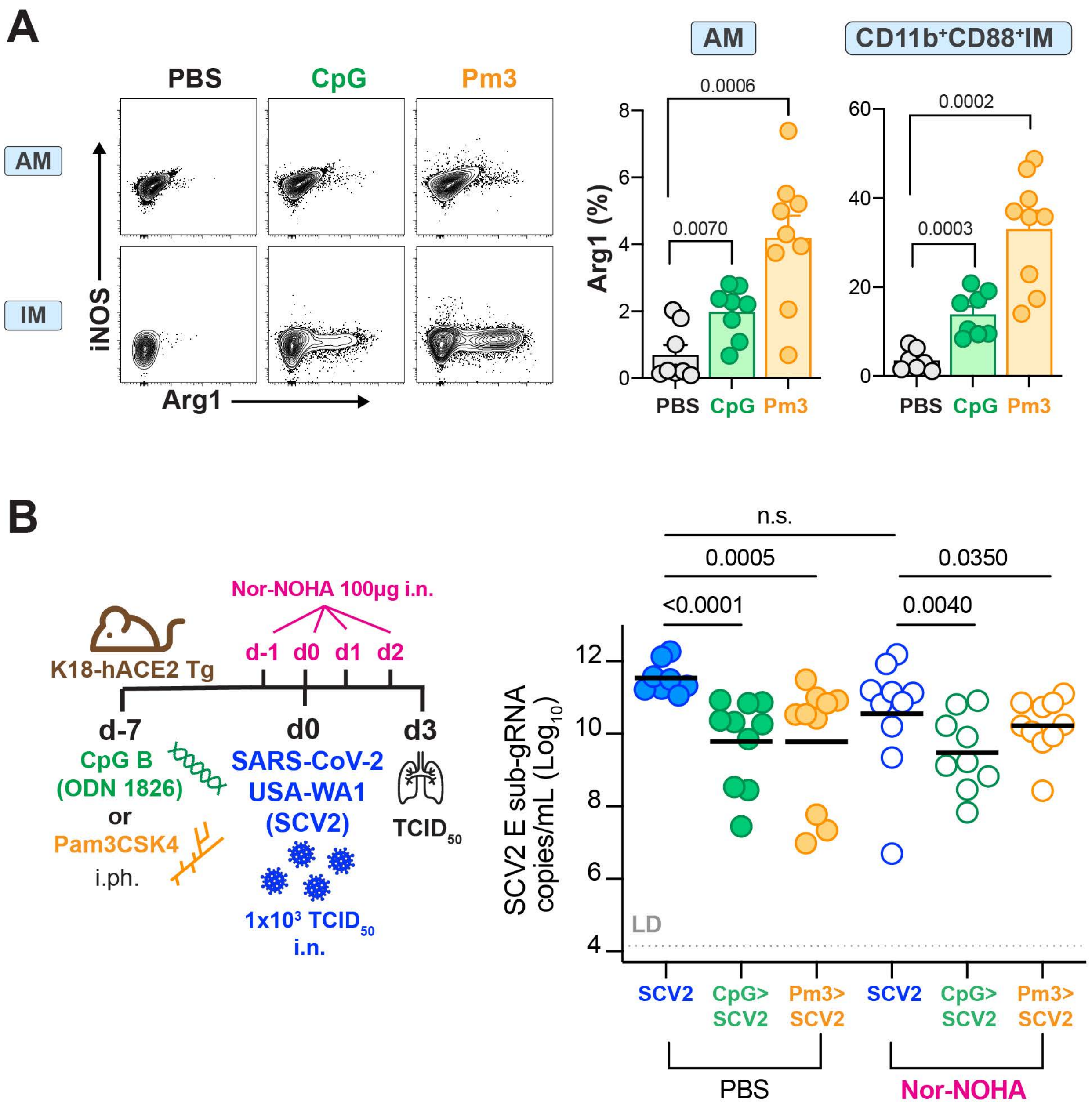

bioRxiv preprint doi: https://doi.org/10.1101/2024.03.27.586885; this version posted March 27, 2024. The copyright holder for this preprint (which was not certified by peer review) is the author/funder. This article is a US Government work. It is not subject to copyright under 17 USC 105 and is also made available for use under a CC0 license.

Baker et al. Figure S5

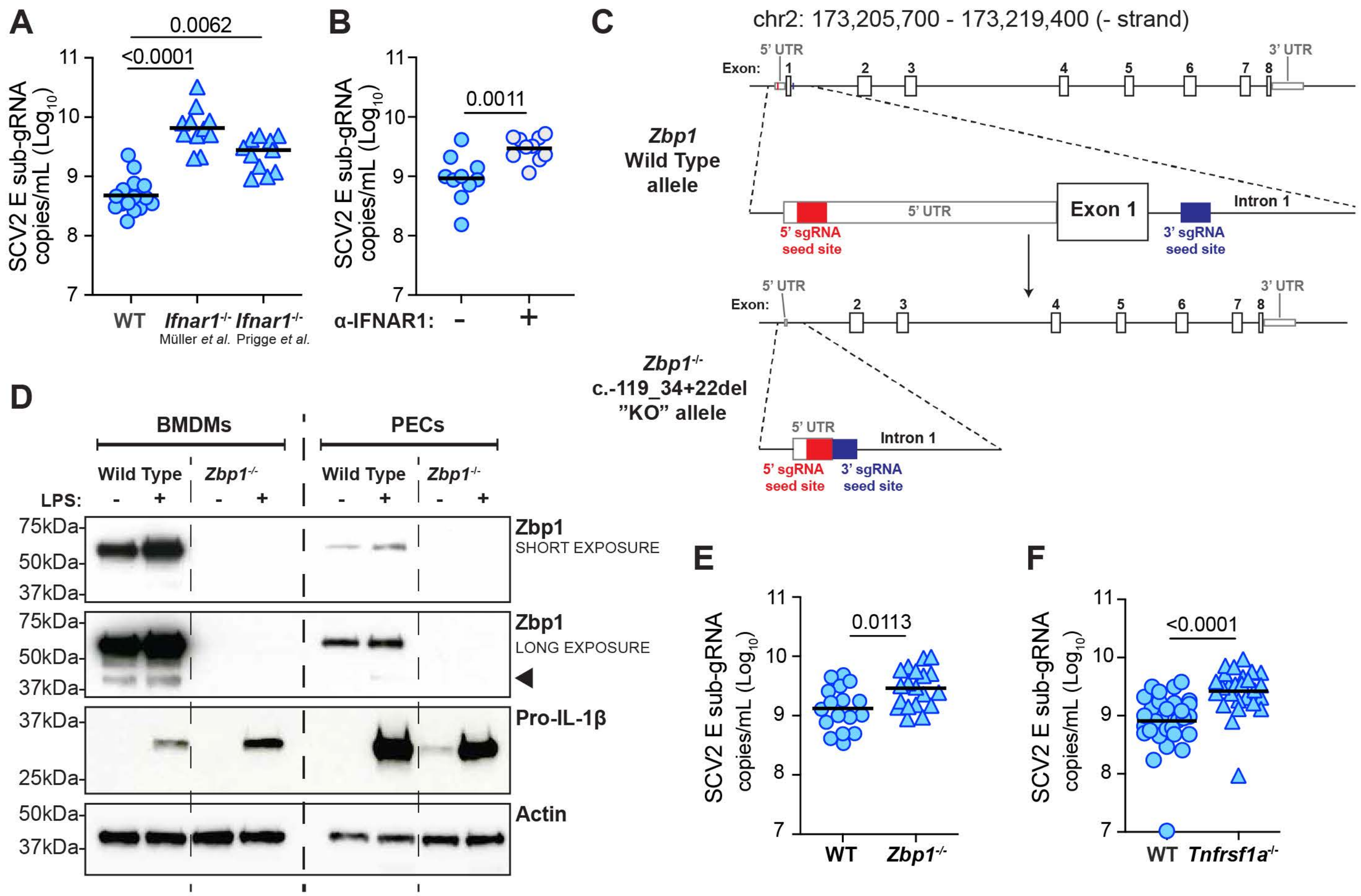

bioRxiv preprint doi: https://doi.org/10.1101/2024.03.27.586885; this version posted March 27, 2024. The copyright holder for this preprint (which was not certified by peer review) is the author/funder. This article is a US Government work. It is not subject to copyright under 17 USC 105 and is also made available for use under a CC0 license.

Baker et al. Figure S6

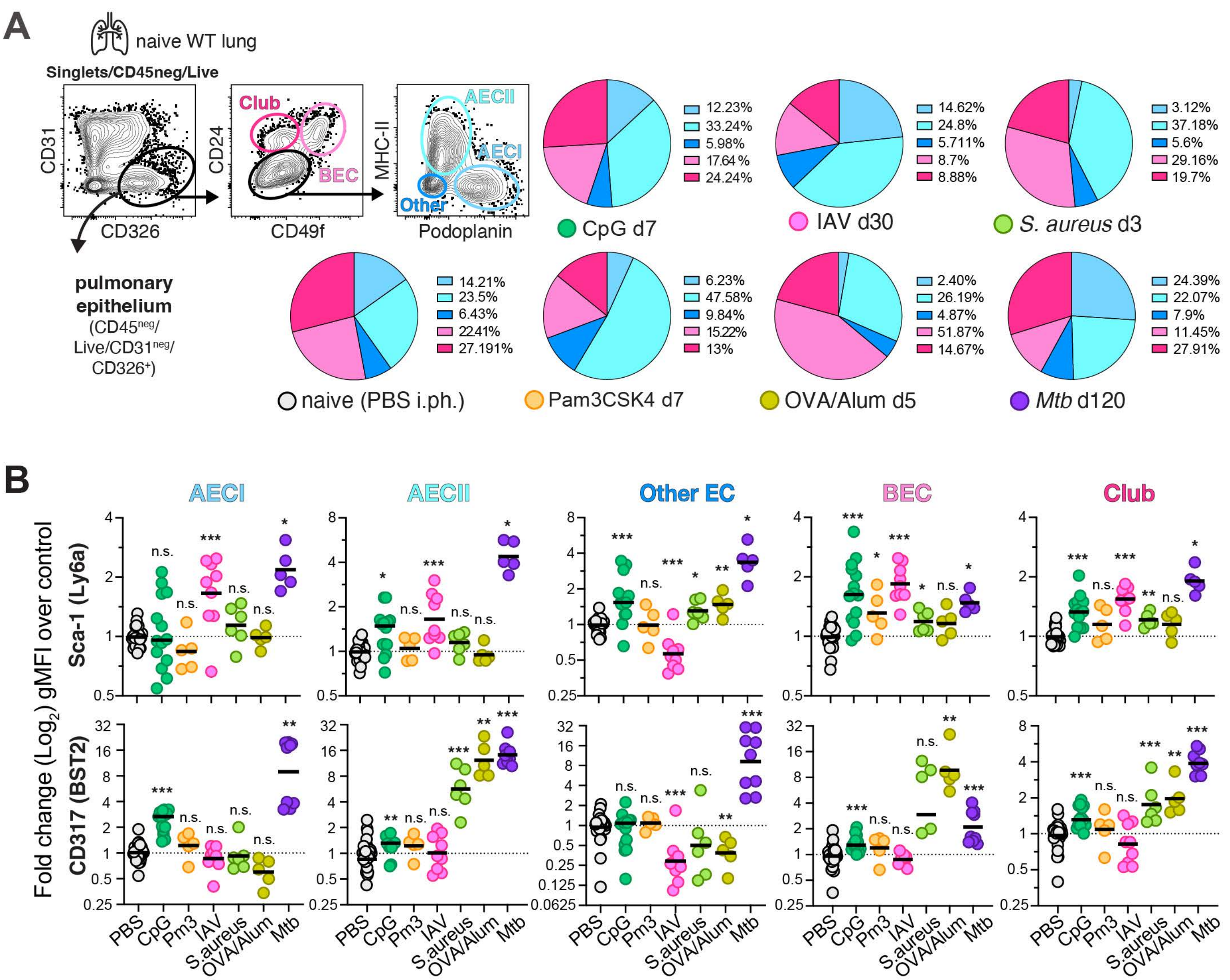

Supplement: Supplement 1 — Figure S1: Recent one-time pulmonary TLR conditioning is sufficient to suppress SCV2 replication in the lung with no changes in gross lung pathology (A) Schematic of WT mice given PBS, 10μg CpG or 50μg Pm3 intrapharyngeally (i.ph.) seven days prior to intranasal (i.n.) infection with 3.5x10^4^ TCID50 SCV2 (B.1.351) and SCV2 viral load in lungs as measured by qPCR for the SCV2 E gene in its sub-genomic form (sub-gRNA) at three days post-infection (3dpi), n= 19-25, data combined from five independent experiments. (B) Schematic of WT mice given either PBS or 10μg CpG i.ph. seven weeks before i.n. infection with 3.5x10^4^ TCID50 SCV2 (B.1.351), and SCV2 viral load in lungs as measured by qPCR for sub-gRNA SCV2 E gene on 3dpi, n= 9-10, data combined from two independent experiments. (C) Representative H&E staining of lung tissue from K18-hACE2 Tg mice given PBS or 10μg CpG i.ph. one week before infection i.n. with 1x10^3^ TCID50 SCV2 (USA-WA1/2020), mice were euthanized 3dpi (scale bars indicate magnification) and percentage of parenchymal enlargement was quantified, n= 8, data combined from two independent experiments. Geometric mean, significance determined by two-tailed Mann-Whitney test, LD= limit of detection, n.s.= not significant. Figure S2: TLR-induced SCV2 restriction is not mediated through reduced ACE2 protein expression and is not reversed by deleting Ccr2 or Trem2. (A) Left: WT mice were administered PBS, CpG or Pm3 intrapharyngeally (i.ph.). Lungs were collected at seven days post treatment and homogenates were assayed for mouse ACE2 by ELISA. Right: K18-hACE2 Tg mice were administered PBS, CpG or Pm3 i.ph., lungs were collected at 10 days post-treatment and homogenates were assayed for human ACE2 by ELISA, n= 3 – 8, data combined from 1 – 2 independent experiments. (B) Trem2−/− or (C) Ccr2−/− mice were given either PBS or 10μg CpG i.ph. seven days prior to being i.n. infected with 3.5x10^4^ TCID50 SCV2 (B.1.351), mice were euthanized 3 days later. Viral loads in l [file NIHPP2024.03.27.586885v1-supplement-1.pdf]
